# Disease-, region- and cell type specific diversity of α-synuclein carboxy terminal truncations in synucleinopathies

**DOI:** 10.1186/s40478-021-01242-2

**Published:** 2021-08-28

**Authors:** Ethan W. Hass, Zachary A. Sorrentino, Yuxing Xia, Grace M. Lloyd, John Q. Trojanowski, Stefan Prokop, Benoit I. Giasson

**Affiliations:** 1 Department of Neuroscience, College of Medicine, University of Florida, BMS J483/CTRND, 1275 Center Drive, Gainesville, FL 32610 USA; 2 Center for Translational Research in Neurodegenerative Disease, College of Medicine, University of Florida, Gainesville, FL 32610 USA; 3 Department of Pathology and Laboratory Medicine, AD Center Core (ADCC), Center for Neurodegenerative Disease Research, PENN) School of Medicine, University of Pennsylvania, Philadelphia, PA 19104 USA; 4 McKnight Brain Institute, College of Medicine, University of Florida, Gainesville, FL 32610 USA; 5 Department of Pathology, College of Medicine, University of Florida, Gainesville, FL 32610 USA

**Keywords:** Carboxy, Inclusions, Lewy body dementia, Multiple system atrophy, α-synuclein, Truncation

## Abstract

Synucleinopathies, including Parkinson’s disease (PD), Lewy body dementia (LBD), Alzheimer’s disease with amygdala restricted Lewy bodies (AD/ALB), and multiple system atrophy (MSA) comprise a spectrum of neurodegenerative disorders characterized by the presence of distinct pathological α-synuclein (αSyn) inclusions. Experimental and pathological studies support the notion that αSyn aggregates contribute to cellular demise and dysfunction with disease progression associated with a prion-like spread of αSyn aggregates via conformational templating. The initiating event(s) and factors that contribute to diverse forms of synucleinopathies remain poorly understood. A major post-translational modification of αSyn associated with pathological inclusions is a diverse array of specific truncations within the carboxy terminal region. While these modifications have been shown experimentally to induce and promote αSyn aggregation, little is known about their disease-, region- and cell type specific distribution. To this end, we generated a series of monoclonal antibodies specific to neo-epitopes in αSyn truncated after residues 103, 115, 119, 122, 125, and 129. Immunocytochemical investigations using these new tools revealed striking differences in the αSyn truncation pattern between different synucleinopathies, brain regions and specific cellular populations. In LBD, neuronal inclusions in the substantia nigra and amygdala were positive for αSyn cleaved after residues 103, 119, 122, and 125, but not 115. In contrast, in the same patients' brain αSyn cleaved at residue 115, as well as 103, 119 and 122 were abundant in the dorsal motor nucleus of the vagus. In patients with AD/ALB, these modifications were only weakly or not detected in amygdala αSyn inclusions. αSyn truncated at residues 103, 115, 119, and 125 was readily present in MSA glial cytoplasmic inclusions, but 122 cleaved αSyn was only weakly or not present. Conversely, MSA neuronal pathology in the pontine nuclei was strongly reactive to the αSyn x-122 neo-epitope but did not display any reactivity for αSyn 103 cleavage. These studies demonstrate significant disease-, region- and cell type specific differences in carboxy terminal αSyn processing associated with pathological inclusions that likely contributes to their distinct strain-like prion properties and promotes the diversity displayed in the degrees of these insidious diseases.

**Supplementary Information:**

The online version contains supplementary material available at 10.1186/s40478-021-01242-2.

## Background

Parkinson’s disease (PD), Lewy body dementia (LBD) and multiple system atrophy (MSA) are characterized by the progressive accumulation of brain intracytoplasmic inclusions comprised of the protein α-synuclein (αSyn) and are therefore collectively termed α-synucleinopathies [11, 21–23, 66]. The definite role for αSyn in the etiology of these diseases was established by the discovery of missense mutations or duplication/triplication of the αSyn gene (*SCNA*) resulting in autosomal-dominant early-onset PD or LBD [2, 10, 16, 34, 36, 37, 46, 49, 51, 56, 68]. Furthermore, numerous in vitro and animal experimental findings, as well as postmortem human studies, support the notion that the aberrant aggregation of αSyn and formation of pathological inclusions can spread throughout the CNS by a prion-like conformational templating mechanism that parallels the insidious nature of these human diseases [8, 27, 64].

The biological mechanisms involved in initiating and promoting the spread of αSyn inclusion pathology that can present with “prion strain-like” properties are still highly debated [8, 27, 64]. While αSyn alterations due to genetic mutations are rare, αSyn species truncated at the carboxy terminus invariantly accumulate to comprise ~ 20% of αSyn within pathological inclusions [1, 3, 9, 33, 39, 44, 57]. Mechanistically, carboxy truncations of αSyn promote both its aggregation and toxicity [57, 60]. Furthermore, the notion that specific αSyn carboxy truncations might be important in disease pathogenesis is underscored by a report that patients with appendectomy are less likely to develop PD, while selective carboxy truncated forms of αSyn can preferentially be generated in the vermiform appendix [35]. The presence of these various forms of truncated αSyn have been identified using mass spectrometry techniques, as well as with antibodies detecting neo-epitopes specific for truncated forms of αSyn (reviewed in [57]). Furthermore, using antibodies targeting the middle region of αSyn, or different amino acid stretches within its carboxy region, we were able to highlight that various forms of αSyn pathology had been underappreciated likely due to modification within this region [25, 58]. However, the investigation of the presence of specific carboxy truncated forms of αSyn at the cellular level can only be performed by immunostaining with antibodies that exclusively react with the precise carboxy truncated forms of αSyn, as this cannot be readily achieved with biochemical methods. Only a limited number of studies have investigated the presence and properties of unique carboxy truncated forms of αSyn within human pathological inclusions due to the limited availability of antibodies specific for these modifications. Previous studies with carboxy truncated specific antibodies have shown that αSyn cleaved after residue 119 is detected in MSA glial cytoplasmic inclusions (GCIs) and Lewy pathology in PD, LBD and incidental Lewy body (LB) disease [1, 38, 50], while reactivity to αSyn cleaved at residues 103 and 122 was detected in Lewy pathology [14, 69]. These previous pathological assessments of human tissue were performed with rabbit antibodies to short peptides corresponding to carboxy truncated forms of αSyn at residues 103, 119 and 122 [1, 14, 38, 50, 69] and some of these antibodies were proprietarily generated by Elan Pharmaceuticals (South San Francisco, CA, USA) [1, 50] and are not currently available.

Given the growing evidence that carboxy truncated forms of αSyn can be involved in the etiology of synucleinopathies [57], herein, we generated and characterized a new series of monoclonal antibodies specific for six of the cleaved forms of αSyn. We use these antibodies to investigate the cellular, neuroanatomical, and disease specificity of these post-translationally modified forms of αSyn within various αSyn pathologies associated with human neurodegenerative diseases.

## Methods

### *Generation of new *α*Syn monoclonal antibodies*

**Fig. 1:**
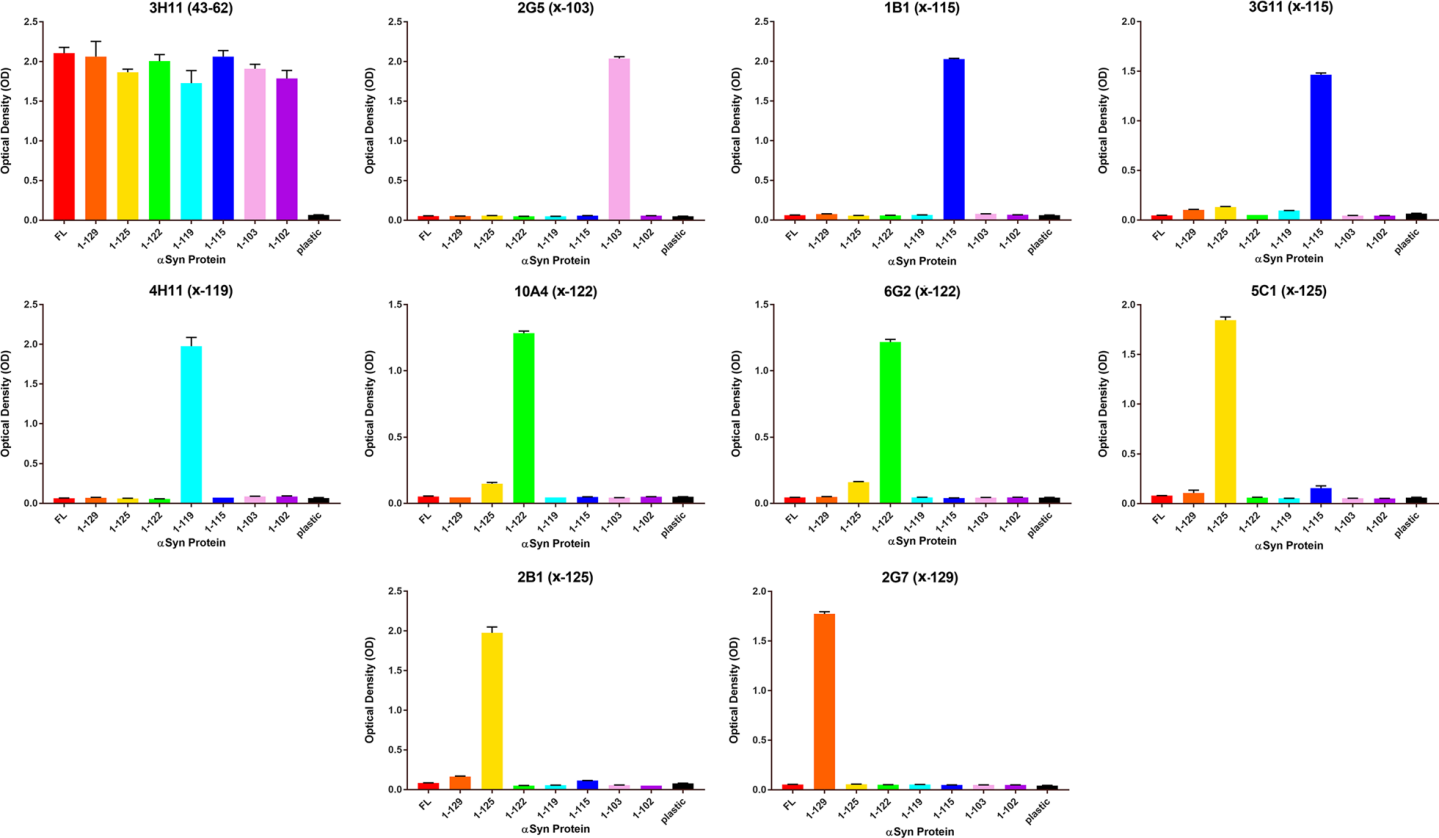
ELISA characterization of the specificity of new monoclonal antibodies to carboxy truncated forms of αSyn. ELISA were performed for all the antibodies as identified above the graphs using 1–102, 1–103, 1–115, 1–119, 1–122, 1–125, 1–129, and full-length (FL; 1–140) recombinant αSyn protein as described in “Material and Methods”. N = 3. The error bar equals standard error of the mean

**Fig. 2:**
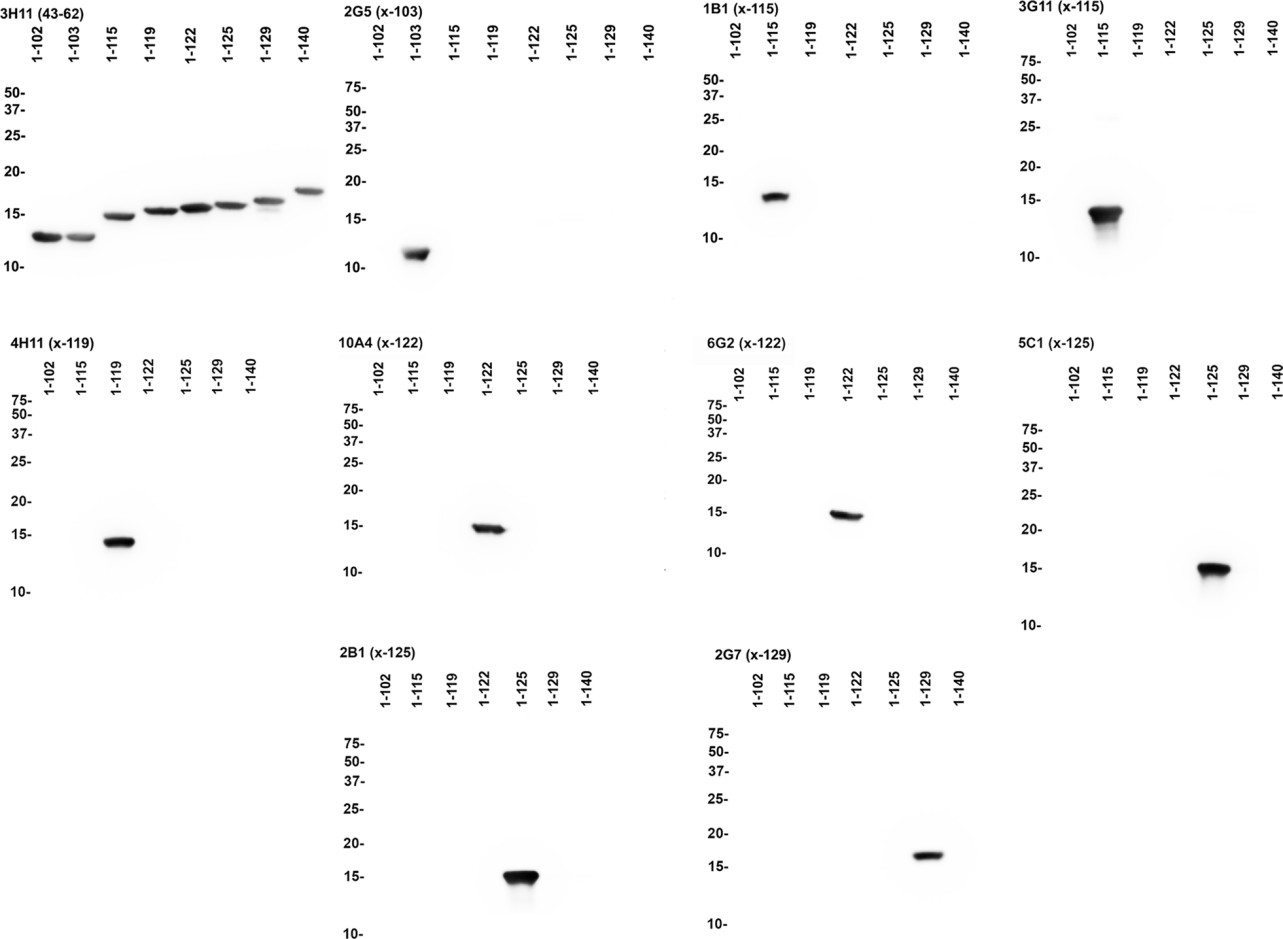
Western blot analyses to demonstrate the specificities of new monoclonal αSyn antibodies. Immunoblot analyses was performed using 1–102, 1–103, 1–115, 1–119, 1–122, 1–125, 1–129, and full-length (FL; 1–140) recombinant αSyn protein as indicated above each lane and as described in “Material and Methods”. 210 ng of recombinant protein was loaded per lane and membranes were probed with the primary antibodies listed above each panel. Relative motilities of molecular mass markers are indicated on the left side of each blot

Synthetic peptides, listed in Table 1, corresponding to different amino acid stretches within the carboxyl terminal region of αSyn were synthesized and purified by GenScript USA, Inc. (Piscataway, NJ). All peptides contained an added Cys residue at the amino terminus that allowed for conjugation to maleimide-activated mariculture keyhole limpet hemocynanin (mcKLH) (Thermo Scientific, Waltham, MA). The peptides conjugated to mcKLH were used to immunize female BALC/c mice (Jackson Laboratory, Bar Harbor, ME) as previously described [53]. All procedures were performed according to the NIH Guide for the Care and Use of Experimental Animals and were approved by the University of Florida Institutional Animal Care and Use Committee. The spleens from the immunized mice were harvested, and the white blood cells were fused with mouse myeloma cells (Sp2/O-Ag14; ATCC, Manassas, VA) as previously described [53]. Following selection with HAT supplement (Sigma Aldrich, St. Louis, MO), all hybridoma clones were initially screened for reactivity by enzyme-linked immunosorbent assay (ELISA) using the respective peptides used for immunization. Specific monoclonal antibodies for each truncated form of αSyn were then identified by ELISA using recombinant full-length αSyn compared to each respective recombinant carboxy truncated form of αSyn. The specificities of each antibody were further confirmed by ELISA and immunoblotting with a series of recombinant carboxy truncated αSyn (see Figs. 1 and 2). Antibody isotypes (Table 1) were determined using a mouse monoclonal isotyping kit (Millipore Sigma, Burlington, MA).

**Table 1:** List of new antibodies described here

Antibody name	Immunization peptide	αSyn residues	Isotype
2G5	CFVKKDQLGKN	94–103	IgG1
1B1	CAGPQEGILED	106–115	IgG1
3G11	CAGPQEGILED	106–115	IgG2B
4H11	CEGILEDMPVD	110–119	IgG1
6G2	CLEDMPVDPDN	113–122	IgG1
10A4	CLEDMPVDPDN	113–122	IgG1
5C1	CDMPVDPDNEAY	115–125	IgG1
2B1	CDMPVDPDNEAY	115–125	IgG1
2G7	CPVDPDNEAYEMPS	117–129	IgG1

Shown are the synthetic peptides used for mouse immunization and their corresponding residues localization in human αSyn. The isotype of each antibody is included

### *Other *α*Syn antibodies*

2H6, 3H11 and 94-3A10 are mouse monoclonal anti-αSyn antibodies with the epitopes including residues 2–21, 43–62 and 130–140, respectively [12, 13].

### *Enzyme-linked immunosorbent assay (ELISA)*

96-well ELISA plates (Corning Life Sciences, Corning, NY) were coated with 100 ng peptide in 100 µL phosphate buffered saline (PBS) per well using the peptide used for immunization (see Table 1). Wells were washed with PBS and blocked with PBS/5% fetal bovine serum (FBS). Primary antibodies were added to blocking solution and incubated at room temperature. After PBS washes, plates were incubated with horseradish peroxidase-conjugated anti-mouse antibody (Jackson Immuno Research Labs, West Grove, PA) in 5% FBS/PBS for an hour. Plates were washed with PBS and 3,3',5,5'-tetramethylbenzidine (TMB substrate, Thermo Fisher Scientific, Waltham, MA) was added to each well. The reactions were stopped by adding 0.5 M HCl and the optical density was measured at 450 nm with a plate reader.

### *Immunohistochemistry of human brain tissue*

Formalin-fixed brain samples of patients with LBD, Alzheimer’s disease with amygdala restricted Lewy bodies (AD/ALB), MSA and controls were provided by the University of Florida Neuromedicine Human Brain and Tissue Bank (UF HBTB) (see Table 1) following institutional regulations. Some of these cases were previously used for other studies [13, 25, 58]. A second set of tissue that was fixed in either formalin or 70% ethanol/150 mM NaCl was obtained from the Center for Neurodegenerative Disease Research (CNDR) tissue bank at the University of Pennsylvania (see Table 2) following institutional regulations. Postmortem diagnoses of LBD, MSA, AD neuropathological change and other changes were made according to current guidelines and criteria proposed by the National Institute of Aging-Alzheimer’s Association [28], the Dementia with Lewy Bodies Consortium [42], and the Neuropathology Working Group on MSA [20]. See Tables 2 and 3 for details on human cases used for this study.

**Table 2:** Summary of cases form the University of Florida Neuromedicine Human Brain and Tissue Bank used in this study

Case	Clinical diagnosis	Primary pathological diagnosis	Secondary pathological diagnosis	Amyloid score (Thal)	Braak	CERAD	Gender	Age	PMI (hrs)
Control 1	Normal	AD low		1	II	none	M	88	4
Control 2	Normal	PART		0	II	none	F	72	4
Control 3	Normal	No significant pathological findings		0	0	none	F	55	12
Control 4	Progressive Myoclonic Epilepsy (Unverricht-Lundborg variant: EPM1)*	No significant pathological findings		0	0	0	M	51	31
FTLD-1	FTLD	FTLD-TDP43		1	0	none	F	67	30
MSA-1	MSA-C	MSA		0	0	none	F	67	4
MSA-2	MSA-P	MSA	AD low	1	I	none	F	60	6
MSA-3	MSA-P	MSA	PART	0	II	none	M	77	18
MSA-4	MSA-C	MSA	AD low; CAA	2	I	sparse	M	71	4
MSA-5	MSA-C	MSA	AD intermediate	2	III	sparse	M	59	4
MSA-6	MSA-P/C	MSA	PART, CAA	0	I	none	F	66	22
MSA-7	MSA-P	MSA		0	0	none	F	66	14
LBD-1	DLB	LBD diffuse neocortical	AD intermediate; CAA	3	IV	moderate	M	67	13
LBD-2	DLB	LBD diffuse neocortical	AD intermediate; CAA; ARTAG	3	III	sparse	F	67	4
LBD-3	AD	LBD diffuse neocortical	AD intermediate; CAA	3	IV	frequent	F	81	15
LBD-4	AD	LBD diffuse neocortical	AD high; CAA	3	V	frequent	F	74	8
LBD-5	AD	LBD diffuse neocortical	AD high; CAA	3	VI	frequent	M	80	21
AD/ALB-1	AD	LBD limbic-transitional	AD intermediate; CAA	2	V	moderate	F	83	9
AD/ALB-2	AD	LBD amygdala	AD high; CAA	3	VI	frequent	M	64	3
AD/ALB-3	AD	LBD amygdala	AD high; CAA	3	VI	frequent	F	67	8

Listed are the clinical and pathological diagnoses, the sex, age at death, amyloid score (Thal), Braak stage and CERAD ratings. *AD* Alzheimer’s disease, *AD/ALB* AD with amygdala restricted Lewy bodies, *ARTAG* aging related tau astrogliopathy, *CAA* cerebral amyloid angiopathy, *DLB* dementia with Lewy body, *FTLD* frontotemporal lobar degeneration, *LATE* limbic-predominant age related TDP-43 encephalopathy, *LBD* Lewy body dementia, *MSA-C* multiple system atrophy with predominant cerebellar ataxia, *MSA-P* multiple system atrophy with predominant Parkinsonism, *PART* primary age-related tauopathy, *PMI* postmortem interval. * *CTSB* mutation

**Table 3:** Summary of cases from the University of Pennsylvania Tissue Bank used in this study

Case	Clinical diagnosis	Pathological diagnosis	Amyloid score (Thal)	Braak	CERAD	Gender	Age	PMI (hrs)
MSA-8	Spinocerebellar ataxia	MSA	1	II	sparse	Female	76	12
MSA-9	MSA-C	MSA	0	0	none	Male	68	8
MSA-10	MSA-C	MSA	0	II	none	Male	72	9
LBD-6	PD	LBD diffuse neocortical	2	III/IV	frequent	Female	82	5
LBD-7	DLB	LBD diffuse neocortical	1	I/II	none	Male	68	15
LBD-8	Corticobasal syndrome	LBD diffuse neocortical	3	I/II	moderate	Male	83	20

Listed are the clinical and pathological diagnoses, the sex, age at death, amyloid score (Thal), Braak stage and CERAD ratings. *DLB* dementia with Lewy body, *LBD* Lewy body dementia, *MSA-C* multiple system atrophy with predominant cerebellar ataxia, *MSA-P* multiple system atrophy with predominant Parkinsonism, *PD* Parkinson’s disease, *PMI* postmortem interval

For immunohistochemistry (IHC), paraffin-embedded tissue on slides were rehydrated in xylene and series of ethanol solutions (100%, 90%, and 70%). For antigen retrieval with most of the αSyn carboxy terminal specific antibodies, tissue sections were placed in a steam bath for 60 min in a solution of modified citrate buffer (Target Retrieval Solution Citrate pH 6; Agilent, Santa Clara, CA). After cooling down using tap water, sections were treated with 70% formic acid for 20 min at room temperature and extensively washed with water. For antibodies 94-3A10 and 4H11, tissue sections were treated with 70% formic acid for 20 min at room temperature and after extensive washing with water, placed in a steam bath for 60 min in a solution of modified citrate buffer (Target Retrieval Solution Citrate pH 6; Agilent, Santa Clara, CA). Endogenous peroxidases were quenched by submerging slides in PBS solutions with 1.5% hydrogen peroxide and 0.005% Triton-X-100. After washing, slides were blocked in 2% FBS/0.1 M Tris, pH 7.6 and were incubated in primary antibody overnight at 4ºC. After washes with 0.1 M Tris, pH 7.6, a mixture of biotinylated secondary antibody (Vector Laboratories; Burlingame, CA) and ImmPRESS polymer secondary antibody (Vector Laboratories; Burlingame, CA) were similarly diluted in block solution and applied to sections for 1 h at room temperature. An avidin–biotin complex (ABC) system (Vectastain ABC Elite kit; Vector Laboratories, Burlingame, CA) was used to enhance detection of the immunocomplexes, which were visualized using the chromogen 3,3′-diaminobenzidine (DAB kit; KPL, Gaithersburg, MD). Tissue sections were counterstained with hematoxylin (Sigma Aldrich, St. Louis, MO). Slides were dehydrated in ethanol solutions (70%, 90%, and 100%) and xylene before they were covered with Cytoseal (Thermo Fisher Scientific, Waltham, MA). Slides were digitally scanned using an Aperio Slide Scanner AT2 instrument (40X magnification; Aperio Technologies Inc., Vista, CA).

### *Semiquantitative assessment of staining intensity and quality for monoclonal antibodies*

Staining intensity and quality were assessed against the baseline of 94-3A10 staining. “Strong” indicates equal staining intensity and quality compared to baseline (best illustrated in Fig. 4 for antibodies 1B1, 10A4, 2G5 and 4H11), while “weak/none” represents absent or very faint staining compared to baseline (best illustrated in Fig. 5 for antibodies 4H11, 2G5 and 10A4). “Moderate” was assigned to staining quality and intensity in between these two extremes, as demonstrated in Fig. 11 for pontine neuronal inclusions in MSA (compared to our previously published data [25]). Assessment of staining intensity and quality was independently conducted by EWH, BIG and SP and the overall consensus score is represented in Table 4.

**Table 4:**
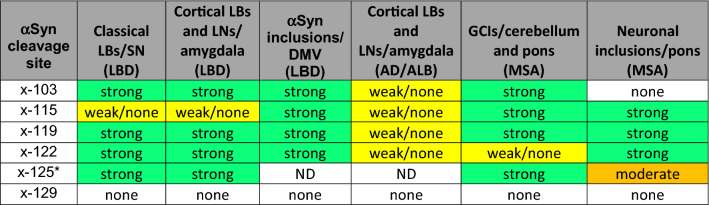
Summary of αSyn pathologies staining with the series for monoclonal antibodies specific for carboxy cleaved αSyn species

*based on staining in ethanol fixed tissue as the x-125 neo-epitope is compromised by formalin fixation. *AD/ALB* AD with amygdala restricted Lewy bodies, *DMV* dorsal motor nucleus of the vagus, *GCIs* glial cytoplasmic inclusions, *LBs* Lewy bodies, *LBD* Lewy body dementia, *LNs* Lewy neurites, *MSA* multiple system atrophy, *SN* substantia nigra, *ND* not determined

### *Sequential biochemical fractionation of human nervous tissue*

Amygdala or temporal cortex adjacent to the amygdala from LBD patients and control without any Lewy pathology were used. Tissues were homogenized with 3 mL per gram of tissue with high salt (HS) buffer (50 mM Tris, pH 7.5, 750 mM NaCl, 20 mM NaF, 5 mM EDTA) with a cocktail of protease inhibitors (1 mM phenylmethylsulfonyl fluoride and 1 mg/ml each of pepstatin, leupeptin, N-tosyl-L-phenylalanyl chloromethyl ketone, N-tosyl-lysine chloromethyl ketone and soybean trypsin inhibitor). The tissue homogenates then underwent sedimentation at 100,000 × g for 30 min and the supernatants were removed and kept as the HS fraction. Pellets were re-extracted in 3 mL per gram of tissue with HS buffer with 1% Triton X-100 (HS/T buffer) and centrifuged at 100,000 × g for 30 min. The supernatants were removed and kept as the HS/T fraction. The pellets were then homogenized in 3 volumes per gram of tissue with HS buffer/1% Triton X-100 with 1 M sucrose and centrifuged at 100,000 × g for 30 min to float the myelin, which was discarded. Pellets were homogenized in 2 mL per gram of tissue with radioimmunoprecipitation assay (RIPA) buffer (50 mM Tris, pH 8.0, 150 mM NaCl, 5 mM EDTA, 1% NP-40, 0.5% sodium deoxycholate, 0.1% SDS) plus protease inhibitors and centrifuged at 100,000 × g for 30 min. Supernatants were removed and kept as the RIPA fraction. Pellets were then homogenized in 1 mL per gram of tissue with 2% SDS/4 M urea by probe sonication, and centrifuged at 100,000 × g for 30 min and supernatant was kept as the SDS/U fractions. Protein concentrations of all fractions were determined by BCA assay using bovine serum albumin (BSA; Pierce, Rockford, IL) as a standard. SDS sample buffer was added to the fractions which were incubated for 10 min at 100 °C (HS and HS/T fractions) or at room temperature (SDS/U fraction only). Equal amounts of protein were resolved by SDS-PAGE and analyzed by immunoblot.

### *Recombinant synuclein proteins*

Full-length (1–140), 1–129, 1–125, 1–122, 1–119, 1–115, 1–103 and 1–102 carboxy truncated human αSyn as well as full-length human β-synuclein (βSyn) and γ-synuclein (γSyn) were expressed using the bacterial expression plasmid pRK172 in BL21 (DE3) E*.coli* (New England Biolabs Inc). Full-length and 1–129, 1–125, 1–122, 1–119, and 1–115 human αSyn recombinant proteins and βSyn and γSyn were purified using size exclusion chromatography followed by Mono Q anion exchange chromatography as previously described [60]. 1–103 and 1–102 carboxy truncated human αSyn were purified using size exclusion chromatography followed by Mono S anion exchange chromatography.

### *Immunoblotting analyses*

Protein samples were resolved by SDS-PAGE on 15% SDS–polyacrylamide gels. The proteins were then electrophoretically transferred onto 0.2 μm pore size nitrocellulose membranes (Bio-Rad, Hercules, CA) in carbonate transfer buffer (10 mM NaHCO_3_, 3 mM Na_2_CO_3_, pH 9.9)[15] with 20% methanol running at constant current of 255 mA for 75 min. Membranes were washed with Tris-buffered saline (TBS), blocked with 5% milk/TBS and incubated overnight at 4 °C with primary antibodies. Following washing, blots were incubated with HRP conjugated goat anti-mouse antibody (Jackson Immuno Research Labs, West Grove, PA) diluted in 5% milk/TBS for 1 h. Following washing, the labeled protein bands were visualized by chemiluminescence using Western Lightning Plus ECL reagents (PerkinElmer Life Sciences, Waltham, MA) and with a GeneGnome XRQ imager (Syngene, Frederick, MD).

## Results

### Generation and characterization of new mouse monoclonal antibodies specific for various carboxy truncated forms of human αSyn

To generate a series of new monoclonal antibodies specific for truncated forms of αSyn, short synthetic peptides with amino acid sequences corresponding to human αSyn ending at residues 103, 115, 119, 122, 125 or 129 (Table 1) were chemically conjugated to mcKLH and used as immunogens. This array of specific cleavage sites in human αSyn was selected based on previous evidence that they were associated with human pathology, occur during the process of cellular transmission or variably affect the propensity of αSyn to aggregate [48, 57, 60]. The hybridomas generated from the fusion of mouse spleen isolated lymphocytes with Sp2/O-Ag14 murine myeloma cells were first screened for antibody secretion by ELISA for reaction with the original peptides used for immunization. The positive antibodies were then screened by ELISA using plates coated with recombinant full-length human αSyn, compared to recombinant human αSyn proteins corresponding to the respective carboxy truncation of each antibody, selecting for antibodies against neo-epitopes that would only be present in the truncated proteins and not full-length proteins.

Monoclonal antibodies specific for each of these carboxy  truncated forms of αSyn (Table 1) were obtained that were further validated by ELISA using the whole spectrum of recombinant carboxy truncated forms of αSyn (Fig. 1), as well as βSyn and γSyn (Additional file 1: Figure S1). These antibodies were very specific for these respective targeted modifications. For example, antibody 2G5 was strongly reactive to αSyn 1–103 but did not react with αSyn 1–102 (Fig. 1). We further validated the specificity of all these antibodies by immunoblotting (Fig. 2) and show that they demonstrate minimal cross-reactivity to other proteins in soluble extracts from human brain (Additional file 1: Figure S2).

### *Labeling pattern of distinct inclusions in synucleinopathies with new monoclonal antibodies specific for carboxy truncated forms *α*Syn*

**Fig. 3:**
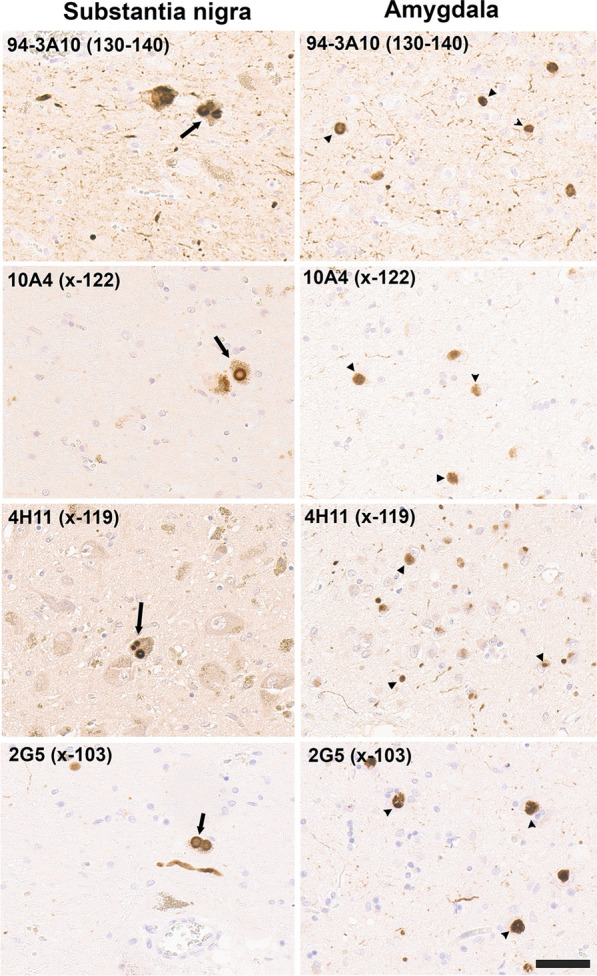
IHC staining of the substantia nigra and amygdala in formalin fixed tissue sections from an LBD patient with antibodies to full length or carboxy truncated forms of αSyn. In the substantia nigra (left panels), antibody 94-3A10 that reacts with amino acid region 130–140 in αSyn and antibodies (10A4, 4H11, and 2G5) specific for carboxy truncated forms of αSyn strongly labeled classic LBs. In the amygdala (right panels), these antibodies also labeled cortical-type LBs and LNs. Tissue sections were stained with the αSyn antibodies indicated in the top left corner. Arrow depicts classical nigral LBs and arrowhead cortical-type LBs. All sections were counterstained with hematoxylin. Scale bar = 50 μm

The new array of monoclonal antibodies specific to various specific forms of carboxy truncated αSyn was used to immunolabel a series of formalin fixed human cases with Lewy body pathology (Table 2). Compared to a αSyn antibody 94-3A10 that reacts with full-length αSyn, antibodies specific for αSyn truncated after residues 122, 119, and 103 extensively labeled classic Lewy bodies in the substantia nigra and cortical-type Lewy bodies and Lewy neurites as shown in the amygdala (Fig. 3). Antibodies specific for αSyn truncated after residues 129, 125, and 115 displayed little to no labeling of these pathologies (data not shown).

**Fig. 4:**
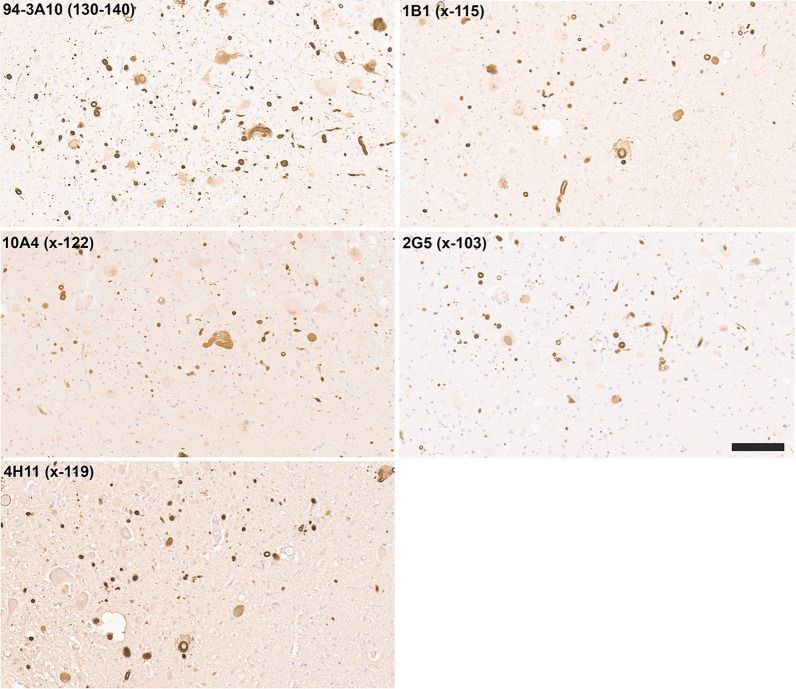
IHC staining within the dorsal motor nucleus of the vagus of formalin fixed tissue sections from an LBD patient with antibodies to full-length or carboxy truncated forms of αSyn. In the DMV, antibody 94-3A10 that reacts with amino acid region 130–140 in αSyn and antibodies (10A4, 4H11, 1B1, and 2G5) specific for carboxy truncated forms of αSyn strongly labeled LBs, LNs, and neuroaxonal spheroids. Tissue sections were stained with the αSyn antibodies indicated in the top left corner. All sections were counterstained with hematoxylin. Scale bar 100 μm

In the dorsal motor nucleus of the vagus (DMV), immunostaining for antibodies specific for αSyn truncated after residues 122, 119, and 103 also revealed strong staining for Lewy bodies and extensive neuroaxonal spheroids (Fig. 4). In contrast to inclusions in the amygdala and substantia nigra, these pathological inclusions were also highlighted with antibodies specific for αSyn truncated after residue 115 (Fig. 4). Antibodies specific for αSyn truncated after residues 129 and 125 displayed little to no labeling of these pathologies in formalin fixed tissue sections (data not shown).

**Fig. 5:**
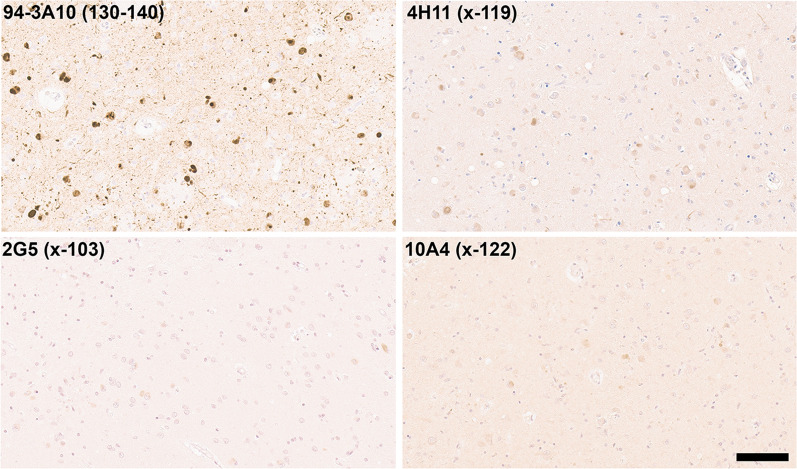
IHC staining of amygdala formalin fixed tissue sections from an AD/ALB brain with antibodies specific for full-length or carboxy truncated forms of αSyn. In the amygdala of AD/ALB, antibody 94-3A10 that reacts with amino acid region 130–140 in αSyn strongly stained Lewy pathology, but antibodies (10A4, 4H11, and 2G5) specific for carboxy truncated forms of αSyn only weakly or did not stain these inclusions. Tissue sections were stained with the αSyn antibodies indicated in the top left corner. All sections were counterstained with hematoxylin. Scale bar 100 μm

In 40–60% of patients with Alzheimer’s disease (AD), Lewy body pathology can be found largely restricted to the amygdala and the closely associated limbic system [24, 45, 65], referred to here as AD/ALB. Staining with our new antibodies for carboxy truncated forms of αSyn demonstrates strong labelling of these neuronal inclusions with antibody 94-3A10, but antibodies specific to αSyn truncated after residues 103 (2G5), 115 (1B1), 119 (4H11) and 122 (10A4) revealed very weak or no staining (Fig. 5; data not shown for 115). Furthermore, inclusions were negative for αSyn cleaved after residues 125 or 129 (data not shown). These findings reveal a distinct pattern of αSyn truncations, distinguishing AD/ALB from LBD (Fig. 3).

**Fig. 6:**
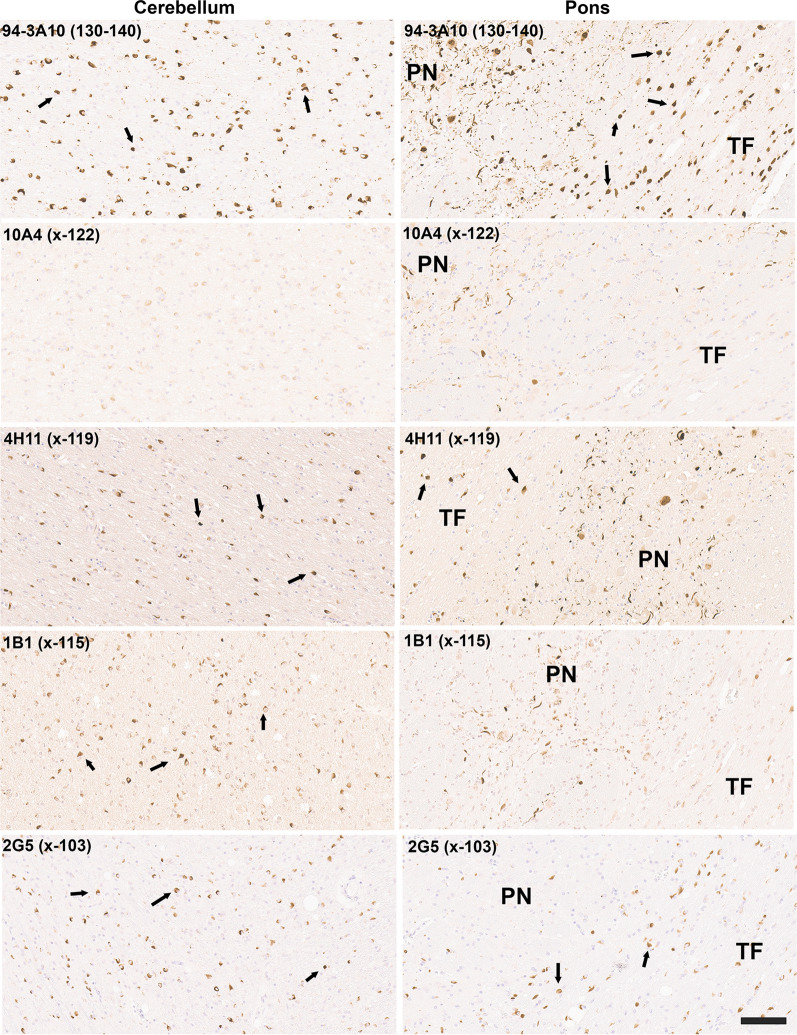
IHC staining of formalin fixed cerebellar and pontine tissue sections from an MSA patient with antibodies to full-length or carboxy truncated form of αSyn. Antibody 94-3A10 that reacts with amino acid region 130–140 in αSyn and antibodies (10A4, 4H11, 1B1 and 2G5) specific for carboxy truncated forms of αSyn were used to stain tissue from the cerebellum white matter region and pons of an MSA patient. In the pons sections, the pontine nuclei (PN) and transverse fibers (TF) regions are identified. Arrows depicts GCIs. Tissue sections were stained with the αSyn antibodies indicated in the top left corner. All sections were counterstained with hematoxylin. Scale bar = 100 μm

GCIs, distinct αSyn inclusions in oligodendrocytes characteristic for MSA were highly reactive with antibodies to αSyn truncated after residues 119, 115, and 103 in both the cerebellum and the pons (Fig. 6). GCIs were also weakly positive for a αSyn x-122 specific antibody in the cerebellum and pons, but this antibody highlighted more strongly neuronal and neuritic pathology in the pontine nuclei [25] (Fig. 6). In contrast, this neuronal pathology in the pontine nuclei was not labeled with αSyn x-103 antibodies, while it showed extensive labelling with antibody 4H11 specific for αSyn x-119. αSyn inclusion pathology in MSA was not labelled with antibodies specific for αSyn x-129 (data not shown) and antibodies to αSyn x-125 only revealed patchy labeling of GCIs (data not shown).

### *Biochemical analyses reveal that *α*Syn x-125 is abundant in aggregated *α*Syn from LBD brain and *α*Syn x-125 pathology is revealed in ethanol fixed tissue.*

**Fig. 7:**
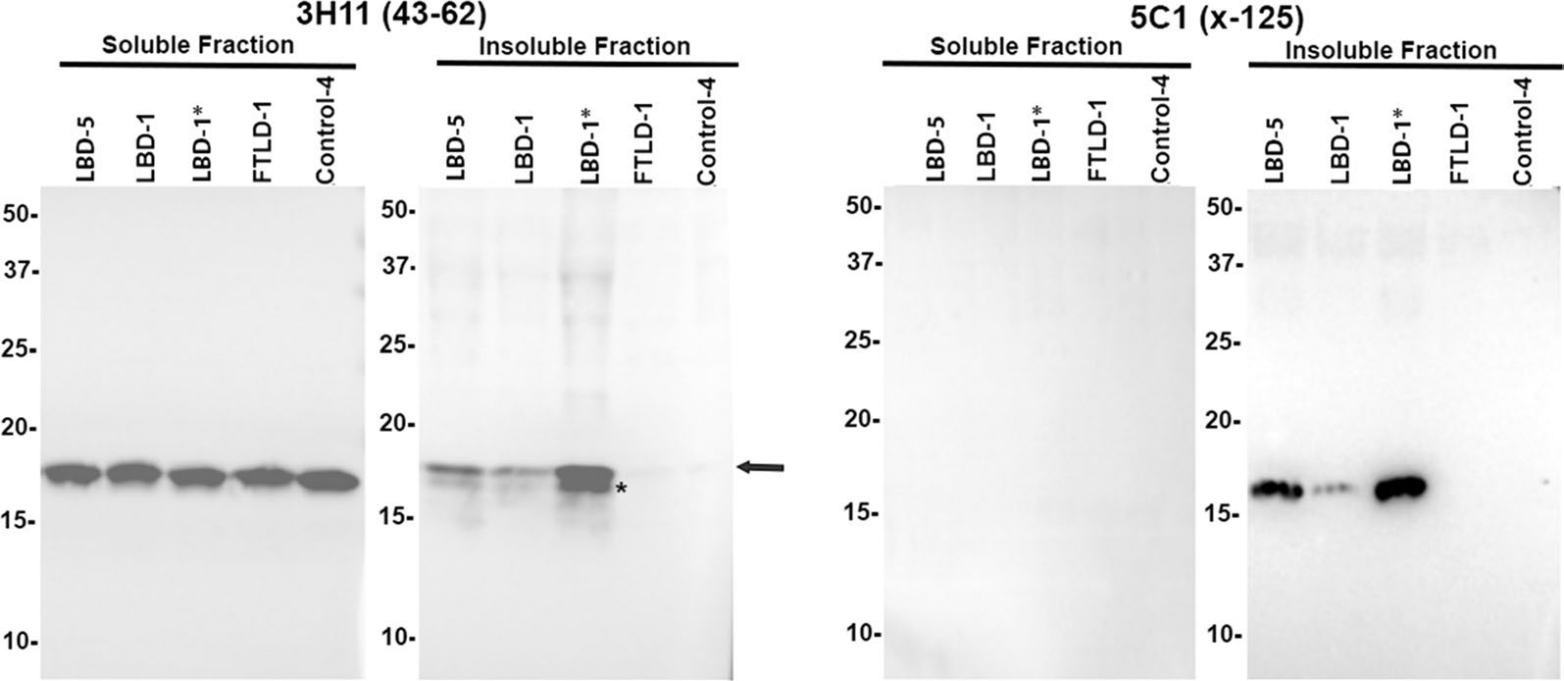
Biochemical fractionation followed by immunoblotting demonstrating that αSyn carboxy truncated at residue 125 is abundant in the brain of LBD patients. LBD temporal cortex adjacent to the amygdala, LBD amygdala (*), and control temporal cortex adjacent to the amygdala were biochemically fractionated as described in “Material and Methods”. 20 ug of protein from the soluble (high salt soluble) and insoluble (SDS/urea) fractions were analyzed by immunoblotting with antibody 3H11 with epitope in the middle of αSyn residues 43–62 and antibody 5C1 specific for αSyn carboxy truncated at residue 125. The relative mobilities of molecular mass protein markers are identified on the left of the blots. Arrow indicates full-length αSyn and asterisk truncated αSyn

**Fig. 8:**
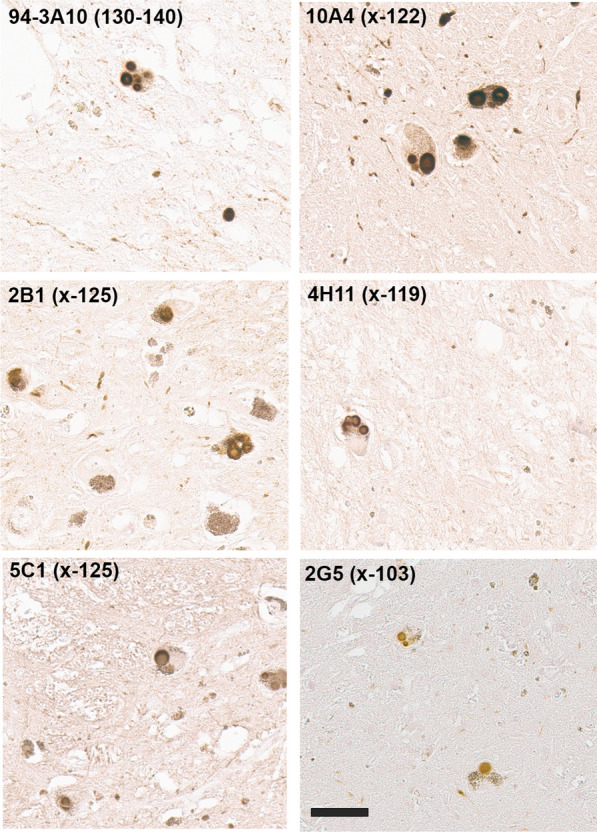
IHC staining of the substantia nigra in ethanol fixed tissue sections from an LBD patient with antibodies to full-length or carboxy truncated forms of αSyn. Substantia nigra tissue sections were labeled with antibody 94-3A10 that reacts with amino acid region 130–140 in αSyn and antibodies (2B1, 5C1, 10A4, 4H11, and 2G5) specific for carboxy truncated forms of αSyn strongly labeled classic LBs. Tissue sections were stained with the αSyn antibodies indicated in the top left corner. All sections were counterstained with hematoxylin. Scale bar = 50 μm

**Fig. 9:**
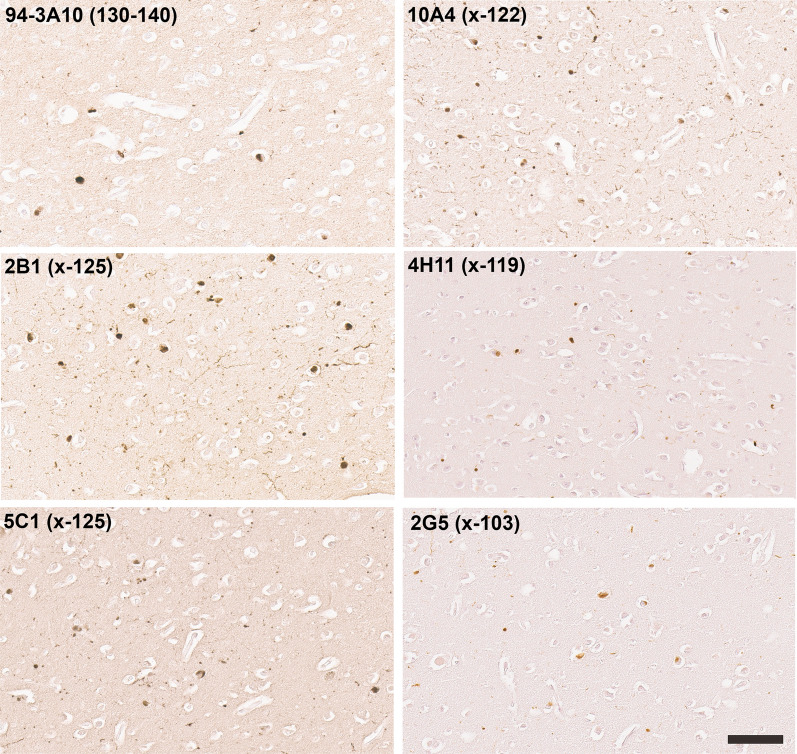
IHC staining of the amygdala in ethanol fixed tissue sections from an LBD patient with antibodies to full-length or carboxy truncated forms of αSyn. Tissue sections from the amygdala were labeled with antibody 94-3A10 that reacts with amino acid region 130–140 in αSyn and antibodies (2B1, 5C1, 10A4, 4H11, and 2G5) specific for carboxy truncated forms of αSyn strongly labeled cortical-type LBs and LNs. Tissue sections were stained with the αSyn antibodies indicated in the top left corner. All sections were counterstained with hematoxylin. Scale bar = 100

**Fig. 10:**
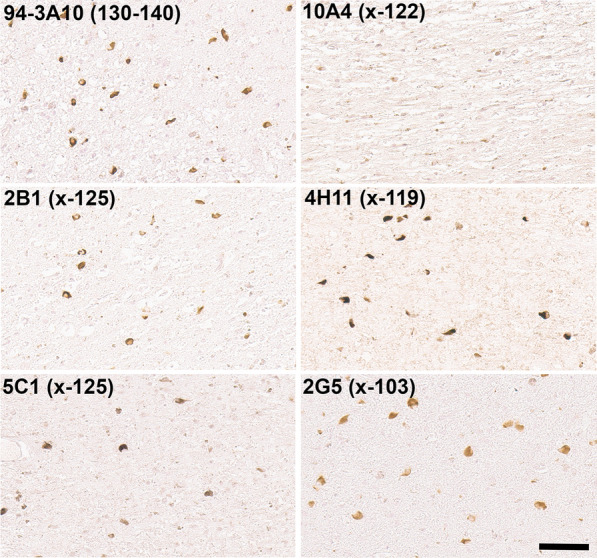
IHC staining of the cerebellum in ethanol fixed tissue sections from an MSA patient with antibodies to full-length or carboxy  truncated forms of αSyn. Cerebellar tissue sections were stained with antibody 94-3A10 that reacts with amino acid region 130–140 in αSyn and antibodies (2B1, 5C1, 10A4, 4H11, and 2G5) specific for carboxy  truncated forms of αSyn. Tissue sections were stained with the αSyn antibodies indicated in the top left corner. All sections were counterstained with hematoxylin. Scale bar = 60 μm

**Fig. 11:**
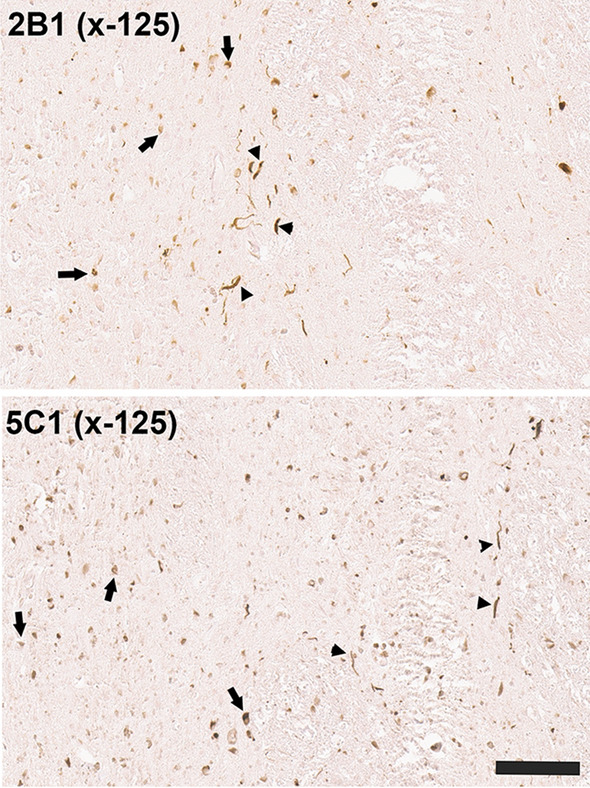
IHC staining of the pons in ethanol fixed tissue sections from an MSA patient with antibodies to αSyn carboxy  truncated at residue 125. Immuno-staining with antibodies 2B1 and 5C1 specific for αSyn carboxy  truncated at residue 125 in the pons region of an MSA patient showing reactivity for both GCIs (arrow) and neuronal neuritic pathology (arrowheads). Tissue sections were stained with the αSyn antibodies indicated in the top left corner. All sections were counterstained with hematoxylin. Scale bar = 100 μm

Biochemical fractionation of brain tissue from patients with LBD followed by immunoblotting demonstrated that detergent-insoluble, aggregated αSyn is only present in patients with Lewy body pathology and not in controls (Fig. 7). However, these analyses also revealed the presence of an abundant protein band that seem to correspond to αSyn truncated at residue 125, which was confirmed by immunoblotting with the specific antibody 5C1 (Fig. 7). In fact, this truncated form of αSyn was only present in the detergent insoluble brain fraction. This finding was perplexing since Lewy body pathology was not labeled by IHC with antibodies 2B1 and 5C1 in formalin fixed paraffin embedded tissue specimens. Given the high degree of specificity of these antibodies to carboxy truncation neo-epitopes we reasoned that they might be sensitive to formalin fixation. Therefore, a series of human postmortem specimens that were fixed in ethanol instead of formalin was obtained for IHC analysis (Table 3). In line with our previous findings (Fig. 3) in formalin fixed tissue, classical LBs in the substantia nigra of ethanol fixed tissue samples were extensively stained with antibodies to αSyn truncated after residues 122, 119, and 103, but also showed strong immunoreactivity with antibodies specific for truncation at residue 125 (Fig. 8). This type of pathology remained negative with antibodies to αSyn x-115 or x-129 even in ethanol fixed tissue (data not shown). Similar findings were obtained for cortical LB and Lewy neurites in ethanol fixed LBD tissue where these were now extensively labeled with antibodies to αSyn x-125 (Fig. 9). In ethanol fixed tissue from MSA patients, GCIs in the cerebellum and pons, as well as neuronal inclusions in the pons were more strongly labeled with αSyn x-125 antibodies 2B1 and 5C1 (Figs. 10 and 11) in contrast to data obtained from formalin fixed tissue (Fig. 6).

## Discussion

αSyn is an abundant neuronal CNS protein but the biological triggers that initiate its aggregation are still controversial. Experimentally, it is clear that the presence of preformed fibrillar αSyn seeds can kick-start this process and that different types of seeds have different potencies that can be propagated with altered properties akin to prion-like strains [8, 27, 64]. αSyn carboxy terminal truncation that can result from varied but specific biological activities is a predominant post-translational modification associated with the formation of αSyn pathological inclusions and this type of modification can dramatically promote the aggregation of αSyn, as the highly negatively charged carboxy terminal region inhibits this process when intact (reviewed in [57]). Additionally, we previously showed that these truncated forms of αSyn can readily form fibrils in conjunctive with full-length αSyn and seed pathology similarly to that of full-length αSyn alone [59, 60], which suggests that the presence of truncated αSyn can initiate and propagate aggregation of full-length αSyn.

**Fig. 12:**
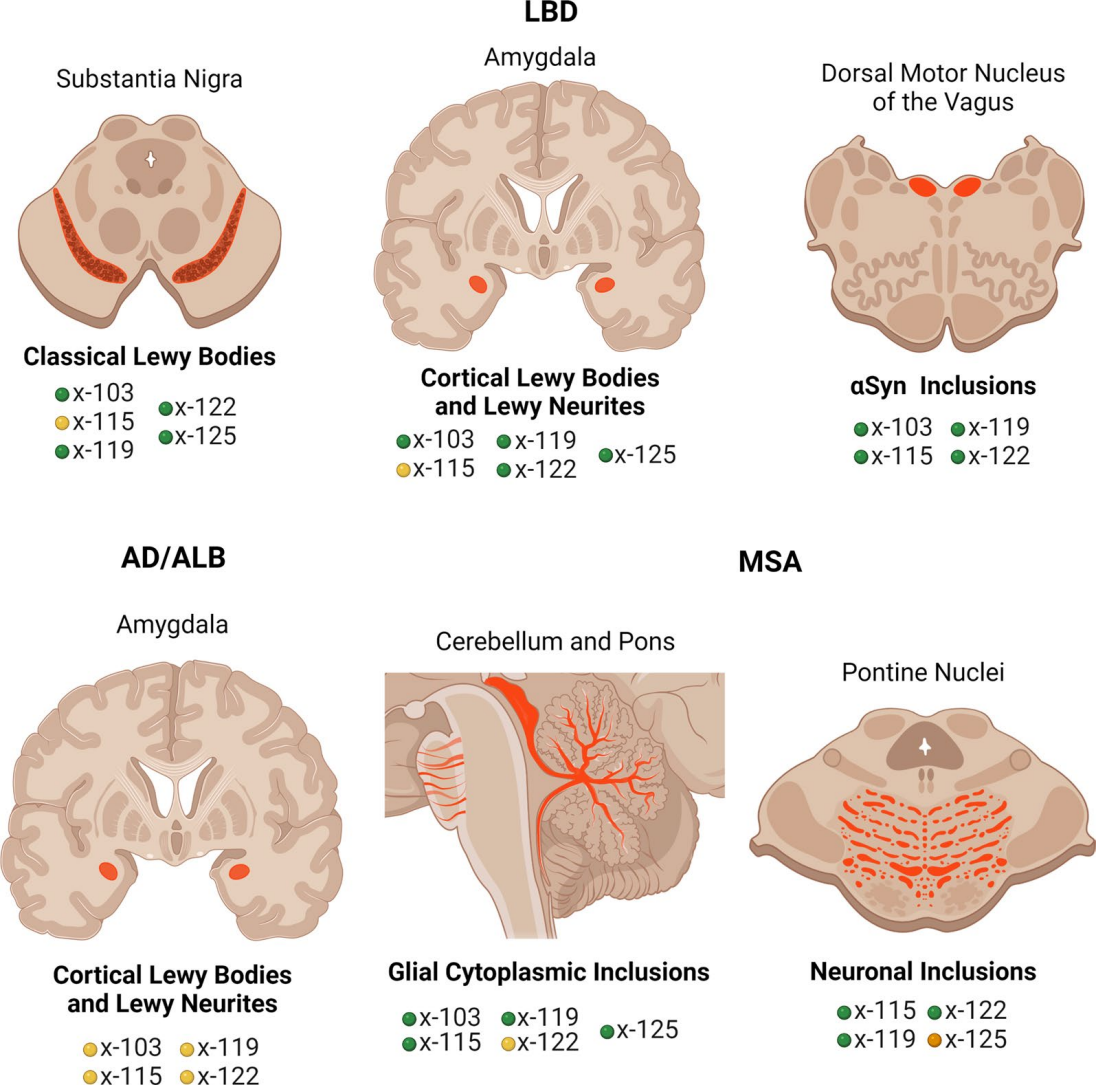
Schematic summary of the pathological findings in the various synucleinopathies assessed for various forms of carboxy truncated αSyn in specific brain regions. Green = strong**,** orange = moderate, yellow = weak/none. Figure was created with Biorender

To provide novel insights on the disease-, regional-, and cell-specific distribution of the major types of αSyn carboxy truncations, a series of specific monoclonal antibodies to αSyn cleaved after residues 103, 115, 119, 122, 125, and 129 was generated that only recognized neo-epitopes resulting from these specific truncations of αSyn. Using these antibodies, new and unexpected region- and disease-specific signatures were revealed (Fig. 12; Table 4). Classical Lewy body pathology and cortical-type LB and LN in the amygdalae of LBD patients were robustly labeled with the antibodies specific to αSyn cleaved after residues 103, 119, and 122 but negative for truncation at residues 115 and 129. By comparison, neuronal αSyn inclusions in the amygdala of AD/ALB patients were only weakly or not labeled with antibodies for αSyn cleaved after residues 103, 119, or 122. Biochemical fractionation in addition revealed αSyn cleaved after residues 125 as a prominent component of inclusions of LBD, which was initially not detected by immunohistochemical staining in formalin fixed tissue sections. Given the potential sensitivity of these neo-epitopes to formalin fixation, we expanded our analysis to an additional set of tissues from cases fixed in ethanol. In these tissues, strong immunoreactivity of LB pathology with 125 truncation specific antibodies was observed in addition to positivity for cleavages after residues 103, 119, and 122 previously detected in formalin fixed tissue. This highlights the importance of tissue preservation and the combination of multiple methodologies when assessing patterns of disease specific post-translational modifications.

The stunning differences in the pattern of carboxy terminal truncations that we observed between αSyn inclusions in the amygdala of LBD and AD/ALB patients suggest that the biological processes involved with these diseases could be distinct. Perhaps, there are differences in the level of the proteinase activities involved in cleaving αSyn or the inclusions are formed by distinct mechanisms such that pathological αSyn in AD/ALB is not exposed to the proteinases. For example, although several non-mutually exclusive cellular mechanisms can be involved in the prion-like transmission of αSyn, many of these pathways involve endosomal-lysosomal compartments [26, 31, 54, 63, 64]. Thus, these processes can be involved in the spread of αSyn pathology in LBD resulting in the carboxy cleavages of αSyn. While in AD/ALB, it is possible that the inclusions are all formed de novo within individual cells without prion-like transmission that may account for the limited neuroanatomical location of αSyn pathology in AD/ALB. Alternatively, it is possible that the biological cleavage of the αSyn carboxy terminus is directly involved in the induction and spread of αSyn and that a more aggressive processing of αSyn in LBD directly contributes to the more ominous neuroanatomical distribution in this disease.

Braak and colleagues suggested that αSyn pathology in PD and LBD can progress via a predictable neuroanatomical distribution and that it might be initiated by some unknown factor(s) in the enteric nervous system that leads to the induction of pathology in the CNS involving specific connections such as the vagal nerve and the DMV [5–7]. Analysis of the DMV in LBD patients revealed abundant αSyn pathology that was intensely stained for antibodies specific for αSyn cleaved after residues 103, 115, 119, and 122. The additional abundant cleavage at residue 115 and the large neuroaxonal spheroids differentiated αSyn pathology in the DMV compared to the amygdala and substantia nigra in the same individuals. Therefore, it is possible that as proposed by Braak and colleagues, there exist some pathogens, perhaps viruses that infect the vagal system and induce the aberrant cleavage of αSyn, precipitating its aggregation and beginning the detrimental seeding and spread of αSyn pathology.

Although αSyn is predominantly a neuronal protein [19, 29, 30, 40], in MSA the defining pathology is abnormal aggregation of αSyn within oligodendrocytes as GCIs [61, 62]. GCIs were strongly reactive for αSyn cleaved after residues 103, 115, 119, and 125, but only displayed very weak reactivity for the 122 cleavage neo-epitope. We recently reported that in the pons of MSA patients there are also abundant neuronal αSyn inclusions within the pontine nuclei that were more extensively revealed by a subset of antibodies with epitopes in the carboxy  terminus of αSyn [25]. This αSyn pathology also presents a differential profile compared to GCIs and neuronal inclusions in LBD, as these are not reactive to αSyn cleaved after 103 but strongly stained for the x-115, x-119, and x-122 neo-epitope and with only modest reactivity for x-125. These differences might reflect relative differences in the enzyme activities responsible for these cleavages in different cellular populations, but could also be due to different prion-like conformational species with differentially present amino acid sequences available for cleavage. For example, proteinase K digestion is often used to assess and define the signature of different prion-like protein strains as different conformers display different accessibility to proteases. Altered cleavage profiles due to structural differences would be consistent with detailed cryo-EM analysis of MSA αSyn fibrils clearly demonstrating that these have different structures than those from LBD [55].

Conversely, experimental modeling studies in mice and in cultured cells implicate that the intracellular environmental milieu of the oligodendrocytes is a driving factor in producing a GCI-type αSyn prion strain(s) with heightened infectivity [47]. So perhaps, the protease activity profiles of oligodendrocytes is distinct from that of neurons, resulting in the differential αSyn cleavage products responsible for the accompanying higher pathogenicity and infectiousness of oligodendrocytic αSyn prion-like strain(s). This notion would be consistent with the observation that levels of αSyn expression are low in oligodendrocytes [43], but that pathology predominantly spreads in these cells in MSA. It is possible that αSyn pathology initially starts in neurons such as in the pontine nuclei where substantial pathology can be present [25] but then it is preferentially processed and propagated in oligodendrocytes despite the much lower expression levels due to a favorable cleavage environment to produce the more potent strains. Aberrant protease activities in MSA patients compared to control could exacerbate this process, and this will be investigated in future studies.

Most of the proteases involved in generating the carboxy  truncated forms of αSyn are found in lysosomes [57]. For some cleavages, more than one protease has been implicated. For example, cathepsin L and asparagine endopeptidase can process αSyn between residues 103 and 104 [41, 69]. It is largely unknown if different cell types and populations in the CNS have different profiles and expression levels of the proteases implicated in cleaving αSyn that could account for some of the differences observed here. Nevertheless, it is interesting that many genetic risk factors associated with α-synucleinopathies, such as glucocerebrosidase insufficiency as well as aging are likely to have impacts on these lysosomal functions and activities that can result in reduced efficiency in the degradation of αSyn and the aberrant accumulation of αSyn carboxy truncated products [4, 17, 32, 52, 67]. Given the intersection between the genetic and pathological findings suggesting a link between abnormal lysosomal activity and αSyn proteolytic processing, future experimental studies will directly investigate the roles carboxy truncated forms of αSyn as initiators and drivers of pathogenesis in the context of lysosomal alterations.

In closing, using a series of novel monoclonal antibodies to neo-epitopes resulting from specific cleavages in the carboxy terminal region of αSyn, we demonstrate significant and surprising disease-, region-, and cell-type specific differences in the profile of αSyn cleavage. Future experimental studies will directly investigate the role of these truncations in the initiation and promotion of disease specific αSyn aggregation using in vivo models. These neo-epitopes are also prominent candidates to develop new and specific CSF and blood biomarker assays. Furthermore, since these epitopes are disease-specific, they can be used to develop future immunotherapies that would not interfere with the activity of normal αSyn as supported by at least one study targeting 122 carboxy truncated αSyn [18].

## Supplementary Information

**Additional file 1: **
**Additional file 1: Figure S1**. ELISA characterization of the specificity of new monoclonal antibodies to carboxy  truncated forms of αSyn to include βSyn and γSyn. ELISA were performed for all the antibodies as identified above the graphs using the specific truncated form of αSyn as well as full-length (FL) recombinant αSyn, βSyn and γSyn proteins as described in “Material and Methods”. *N* = 4. The error bar equals standard error of the mean.
**Additional file 1: Figure S2**. Immunoblotting demonstrating the specificity of αSyn carboxy truncated antibodies using soluble brain lysates. SDS–polyacrylamide gels were loaded with 10 ng of each respective αSyn proteins as indicated above or 20 ug of protein from the soluble (high salt soluble) fractions from the temporal cortex adjacent to the amygdala from the individuals indicated. Immunoblots were probed with the antibodies labelled above each panel.

## Data Availability

All data generated or analyzed during this study are included in this published article and its supplementary information files.

## Abbreviations

**AD**: Alzheimer’s disease
**AD/ALB**: Alzheimer’s disease with amygdala restricted Lewy bodies
**ARTAG**: Aging related tau astrogliopathy
**αSyn**: α-synuclein
**βSyn**: β-synuclein
**CNDR**: Center for Neurodegenerative Disease Research
**DAB**: 3,3′-diaminobenzidine
**DLB**: Dementia with Lewy body
**DMV**: Dorsal motor nucleus of the vagus
**ELISA**: Enzyme-linked immunosorbent assay
**FBS**: Fetal bovine serum
**FTLD**: Frontotemporal lobar degeneration
**γSyn**: γ-synuclein
**GCIs**: Glial cytoplasmic inclusions
**HS**: High salt
**HS/T buffer**: HS buffer with 1% Triton X-100
**IHC**: Immunohistochemistry
**LATE**: Limbic-predominant age related TDP-43 encephalopathy
**LB**: Lewy body
**LBD**: Lewy body dementia
**LNs**: Lewy neurites
**mcKLH**: Mariculture keyhole limpet hemocynanin
**MSA**: Multiple system atrophy
**MSA-C**: Multiple system atrophy with predominant cerebellar ataxia
**MSA-P**: Multiple system atrophy with predominant Parkinsonism
**ND**: Not determined.
**PART**: Primary age-related tauopathy
**PBS**: Phosphate buffered saline
**PD**: Parkinson’s disease
**RIPA**: Radioimmunoprecipitation assay
**PMI**: Postmortem interval
**SN**: Substantia nigra
**TMB**: 3,3',5,5'-tetramethylbenzidine
**UF HBTB**: University of Florida Neuromedicine Human Brain and Tissue Bank
