# Supplementary material for: Disease-, region- and cell type specific diversity of α-synuclein carboxy terminal truncations in synucleinopathies
Source: Acta Neuropathol Commun. 2021 Aug 28;9:146. doi: 10.1186/s40478-021-01242-2 (PMC8403399; doi:10.1186/s40478-021-01242-2)
Supplement: Supplementary file 1 — Additional file 1: Additional file 1: Figure S1. ELISA characterization of the specificity of new monoclonal antibodies to carboxy truncated forms of αSyn to include βSyn and γSyn. ELISA were performed for all the antibodies as identified above the graphs using the specific truncated form of αSyn as well as full-length (FL) recombinant αSyn, βSyn and γSyn proteins as described in “Material and Methods”. N = 4. The error bar equals standard error of the mean. Additional file 1: Figure S2. Immunoblotting demonstrating the specificity of αSyn carboxy truncated antibodies using soluble brain lysates. SDS–polyacrylamide gels were loaded with 10 ng of each respective αSyn proteins as indicated above or 20 ug of protein from the soluble (high salt soluble) fractions from the temporal cortex adjacent to the amygdala from the individuals indicated. Immunoblots were probed with the antibodies labelled above each panel. [file 40478_2021_1242_MOESM1_ESM.pdf]

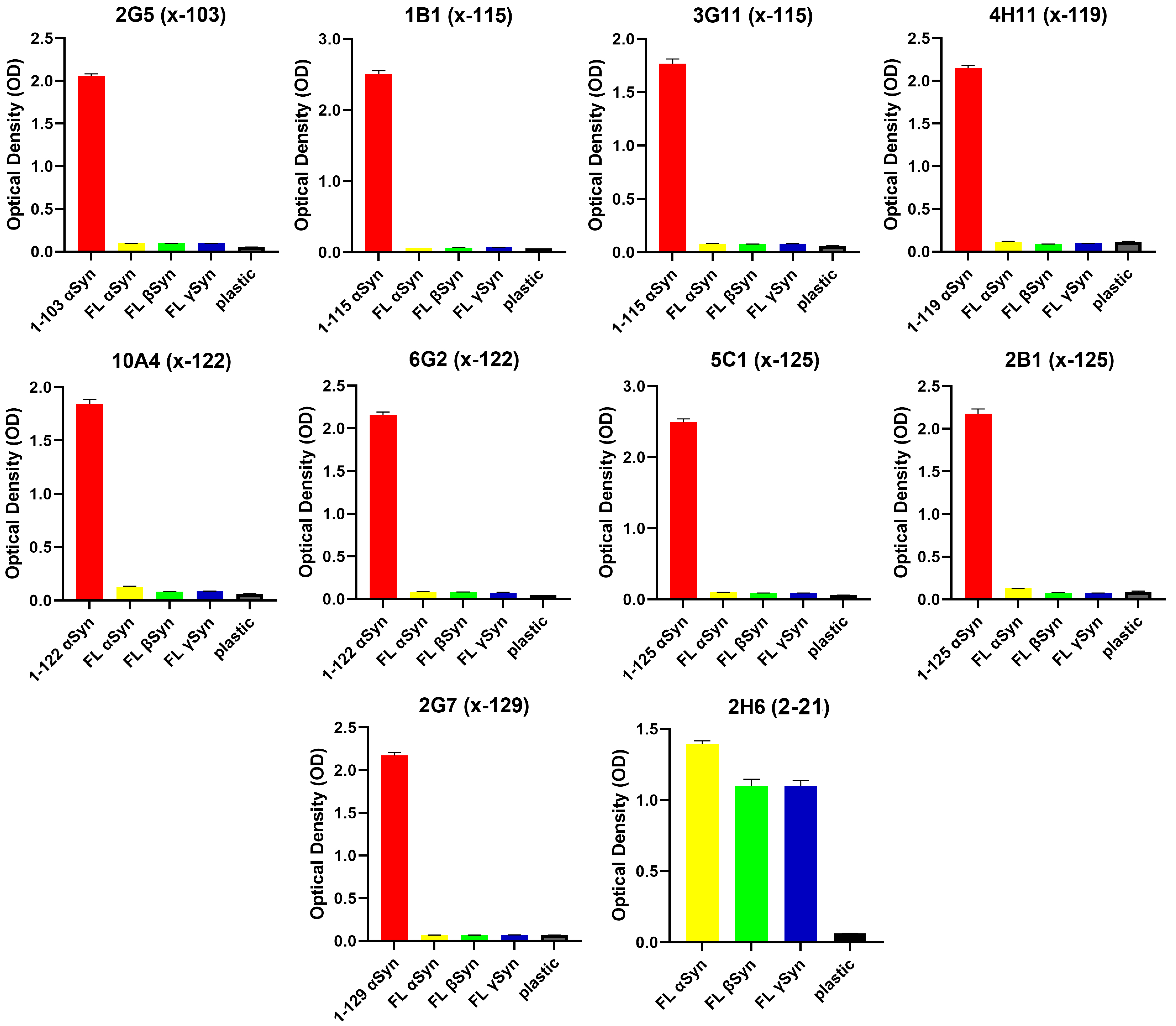

Supplemental Figure 1

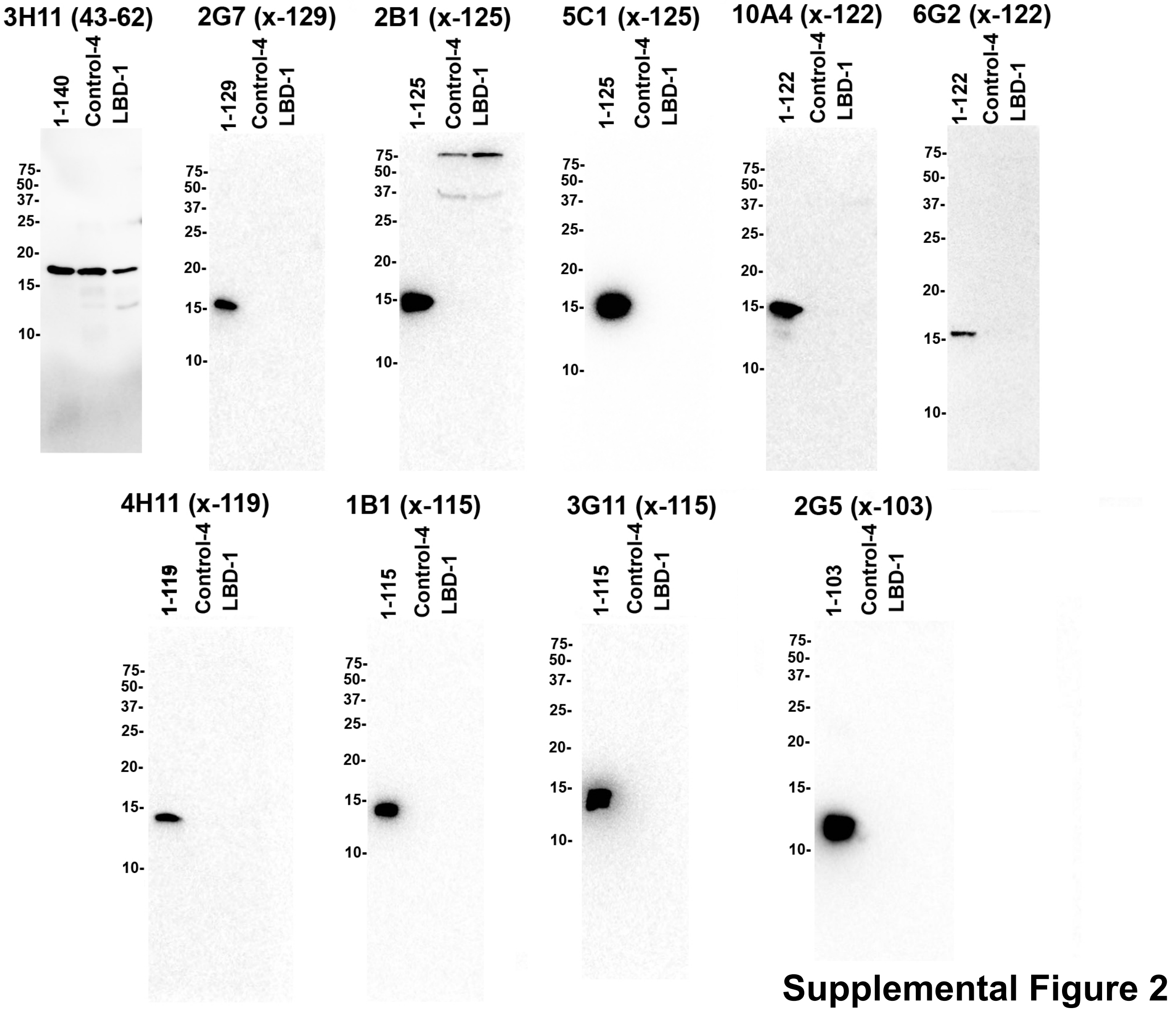

Supplemental Figure 2

Antibody name and preparation type	Dilution for ELISA	Dilution for Western blotting	Dilution for IHC
2G5 (TC supernatant)	1:2	1:2	none
1B1 (TC supernatant)	1:2	1:2	1:10
3G11 (TC supernatant)	1:2	1:2	none
4H11 (TC supernatant)	1:2	1:2	none
6G2 (TC supernatant)	1:2	1:2	none
10A4 (TC supernatant)	1:2	1:2	none
5C1 (TC supernatant)	1:2	1:2	none
2B1 (TC supernatant)	1:2	1:2	none
2G9 (TC supernatant)	1:2	1:2	none
3H11 (ascites fluid)	1:5000	1:1000	NA
94-3A10 (ascites fluid)	NA	NA	1:10,000
2H6 (ascites fluid)	1:2000	NA	NA

Supplemental Table 1. Dilution of Antibody Used. TC, tissue culture media.
